# Determining patients with spinal metastases suitable for surgical intervention: A cost‐effective analysis

**DOI:** 10.1002/cam4.6576

**Published:** 2023-09-25

**Authors:** Hsiang‐Chieh Hsieh, Hung‐Kuan Yen, Ting‐En Tseng, Yu‐Ting Pan, Min‐Tsun Liao, Shau‐Huai Fu, Mao‐Hsu Yen, Fu‐Shan Jaw, Wei‐Hsin Lin, Ming‐Hsiao Hu, Shu‐Hua Yang, Olivier Q. Groot, Andrew J. Schoenfeld

**Affiliations:** ^1^ Institute of Biomedical Engineering, National Taiwan University Taipei Taiwan; ^2^ Department of Orthopaedic Surgery National Taiwan University Hospital Taipei Taiwan; ^3^ Department of Orthopaedic Surgery National Taiwan University Hospital Hsinchu Taiwan; ^4^ Department of Medical Education National Taiwan University Hospital Hsinchu Taiwan; ^5^ Department of Medical Education National Taiwan University Hospital Taipei Taiwan; ^6^ Division of Cardiology, Department of Internal Medicine National Taiwan University Hospital Hsinchu Taiwan; ^7^ Department of Orthopaedic Surgery National Taiwan University Hospital Douliu Taiwan; ^8^ Department of Computer Science and Engineering National Taiwan Ocean University Keelung Taiwan; ^9^ Department of Orthopaedics, College of medicine, National Taiwan University Taipei Taiwan; ^10^ Department of Orthopaedic Surgery Massachusetts General Hospital, Harvard Medical School Boston Massachusetts USA; ^11^ Department of Orthopaedics University Medical Center Utrecht Utrecht Netherlands; ^12^ Department of Orthopaedic Surgery Brigham and Women's Hospital, Harvard Medical School Boston Massachusetts USA

**Keywords:** cost‐effective analysis, neoplasm metastasis, radiation oncology, spine, surgical oncology

## Abstract

**Background:**

Both nonoperative and operative treatments for spinal metastasis are expensive interventions. Patients' expected 3‐month survival is believed to be a key factor to determine the most suitable treatment. However, to the best of our knowledge, no previous study lends support to the hypothesis. We sought to determine the cost‐effectiveness of operative and nonoperative interventions, stratified by patients' predicted probability of 3‐month survival.

**Methods:**

A Markov model with four defined health states was used to estimate the quality‐adjusted life years (QALYs) and costs for operative intervention with postoperative radiotherapy and radiotherapy alone (palliative low‐dose external beam radiotherapy) of spine metastases. Transition probabilities for the model, including the risks of mortality and functional deterioration, were obtained from secondary and our institutional data. Willingness to pay thresholds were prespecified at $100,000 and $150,000. The analyses were censored after 5‐year simulation from a health system perspective and discounted outcomes at 3% per year. Sensitivity analyses were conducted to test the robustness of the study design.

**Results:**

The incremental cost‐effectiveness ratios were $140,907 per QALY for patients with a 3‐month survival probability >50%, $3,178,510 per QALY for patients with a 3‐month survival probability <50%, and $168,385 per QALY for patients with independent ambulatory and 3‐month survival probability >50%.

**Conclusions:**

This study emphasizes the need to choose patients carefully and estimate preoperative survival for those with spinal metastases. In addition to reaffirming previous research regarding the influence of ambulatory status on cost‐effectiveness, our study goes a step further by highlighting that operative intervention with postoperative radiotherapy could be more cost‐effective than radiotherapy alone for patients with a better survival outlook. Accurate survival prediction tools and larger future studies could offer more detailed insights for clinical decisions.

## 1 INTRODUCTION

Management of spinal metastasis is challenging for both medical and orthopedic oncologists. The short life expectancy, severe back pain, and compromised quality of life should be carefully evaluated when tailoring treatment plans. Historically, spine surgeons expected a postoperative survival of at least 3 months to help select suitable candidates for operative intervention in spinal metastasis.^1^, ^2^ Otherwise, radiotherapy should be considered for symptom relief.^3^ The rationale is that only patients with better prognosis have enough time to recover from such major operations. Unfortunately, to the best of our knowledge, no previous cost‐effective analysis lends support to the hypothesis.

In real‐world practice, patients' life expectancy cannot be ascertained in advance. Relying on clinical judgment in oncology patients is reportedly poor.^4^ Utilizing a variety of clinical factors, several survival prediction models (SPM) have been developed to preoperatively estimate prognosis.^5^, ^6^ Among them, the SORG Orthopaedic Research Group machine learning algorithm (SORG‐MLA) provides superior discriminatory ability and clinical benefit.^7^ Although its generalizability was successfully tested in five international institutions,^1^, ^8^, ^9^, ^10^, ^11^ none of the studies discussed its utility in regard to medical economics. Therefore, in this study, we sought to use the SORG‐MLA as a default guide for the clinical decision.

We sought to perform a nuanced cost‐effective analysis that incorporated the survival probability estimates of the SORG‐MLA in models that considered operative management and palliative low‐dose external beam radiotherapy (EBRT) for patients in two different groups: (1) 3‐month survival probability >50%, (2) 3‐month survival probability <50%. We hypothesized that operative treatment is only cost‐effective in the former population but not in the latter population.

## 2 MATERIALS AND METHODS

### 2.1 Study design

This single‐center study followed the Consolidated Health Economic Evaluation Reporting Standards (CHEERS).^12^ The medical records review was approved by the institution's research ethics committee (202105108RINC). The patient's consent was waived due to the retrospective nature of this study.

### 2.2 Populations under considerations and strategies

We considered two populations: (1) 3‐month survival probability >50% and (2) 3‐month survival probability <50%. In both populations, we modeled a patient with metastatic epidural canal compromise at T9 and T10 from a radiosensitive tumor (e.g., breast cancer, cervical cancer, etc.).^13^ Two therapeutic strategies, namely radiotherapy alone and operative intervention with postoperative radiotherapy, were considered in this study. Operative intervention included surgery, which consisted of a 2‐level posterolateral decompression and partial corpectomy with interbody cage, autograft, and 6‐level posterior instrumented fusion. Palliative low‐dose EBRT, namely conventional radiation therapy of 30 Gy within 10 fractions, was the radiotherapy modality used for all patients included in this study.^13^, ^14^

### 2.3 Outcomes

The primary outcome is the differences in costs divided by the differences in quality‐adjusted life years (QALYs), namely the incremental cost‐effectiveness ratio (ICER), between the two strategies. The willingness to pay (WTP) thresholds, which quantifies the maximal cost that society is willing to spend for each additional QALY gained, are prespecified set at $100,000 and $150,000, which are commonly used in the United States.^15^, ^16^ The treatment is only considered cost‐effective if the ICERs are below the prespecified WTP thresholds.^15^

Secondary outcomes include lifetime direct medical costs and QALYs. Quality‐adjusted life years was based on a previously published EuroQol 5‐dimension (EQ5D) survey^17^ and converted from the corresponding utility values.^15^ The estimation was associated with the patient's health status, including revision surgery, radiotherapy failure (the need for a surgical intervention for individuals initially managed nonoperatively), posttreatment complications, and mortality.^17^ Published data were referenced to derive the therapeutic efficacy,^2^, ^16^, ^18^, ^19^ 30‐day mortality rate,^2^, ^17^, ^18^, ^19^, ^20^, ^21^ complication rate,^14^, ^17^, ^20^, ^22^, ^23^ and the other transition probabilities that occurred infrequently (Table S1).^2^, ^17^, ^19^, ^22^, ^23^, ^24^, ^25^, ^26^, ^27^, ^28^, ^29^, ^30^ Monthly mortality rate of each subgroup was derived from our own cohort, which is adjusted from the one‐year all‐cause mortality rate.^3^ The 2019 Medicare fee schedule, which was consistent with the cohort enrollment period, was referenced to calculate the costs of operative and nonoperative treatments.^31^ The costs of prescription medications, outpatient physician visits, emergency room encounters, hospital admission, imaging, anesthesia and surgeon fees, posttreatment care and evaluations, cost for treatment of complications, posttreatment rehabilitation, durable medical equipment, home nursing care, and hospice care were included. The frequency of resource use for each category was determined from expert consultation.^17^ A 3% annual adjustment was applied for costs and quality of life.^15^, ^17^

### 2.4 Patient selection

Adult patients with spinal metastasis who were treated with surgery and/or radiotherapy between 2010 and 2018 at a tertiary center (*n* = 2768) were retrospectively included.^3^ The median age was 61 (interquartile range [IQR], 53–70), and 45% (1238/2768) were female. 22% of the patients (617/2768) received surgical intervention for spinal metastasis, and the remaining 78% (2151/2768) were treated with radiotherapy. The median Karnofsky performance scale was 70 (IQR, 70–80). Only 5% of patients (140/2768) were nonambulatory at presentation, which was prospectively recorded. The ambulatory patients' 3‐month survival rate was 75.7% (1989/2628) and one‐year survival rate was 40.1% (1054/2628). The nonambulatory patients' 3‐month survival rate was 70.0% (98/140) and one‐year survival rate was 29.3% (41/140).

### 2.5 Variables

The following variables were collected for the SORG‐MLA prediction^1^: body mass index, Eastern Cooperative Oncology Group (ECOG) performance status, histology of primary tumor,^32^ visceral metastases, brain metastases, three or more spine metastases, previous systemic therapy, any Charlson comorbidity other than metastatic disease, American Spinal Injury Association (ASIA) Impairment Scale as well as ambulatory status at presentation, and preoperative laboratory values (white blood cell count, hemoglobin, platelet count, absolute lymphocyte and neutrophil count, albumin, alkaline phosphatase, creatinine, and international normalized ratio). The patients' predicted 3‐month survival probabilities were manually derived from the openly accessible web application (https://sorg‐apps.shinyapps.io/spinemetssurvival/).

### 2.6 Statistical analysis

**FIGURE 1:**
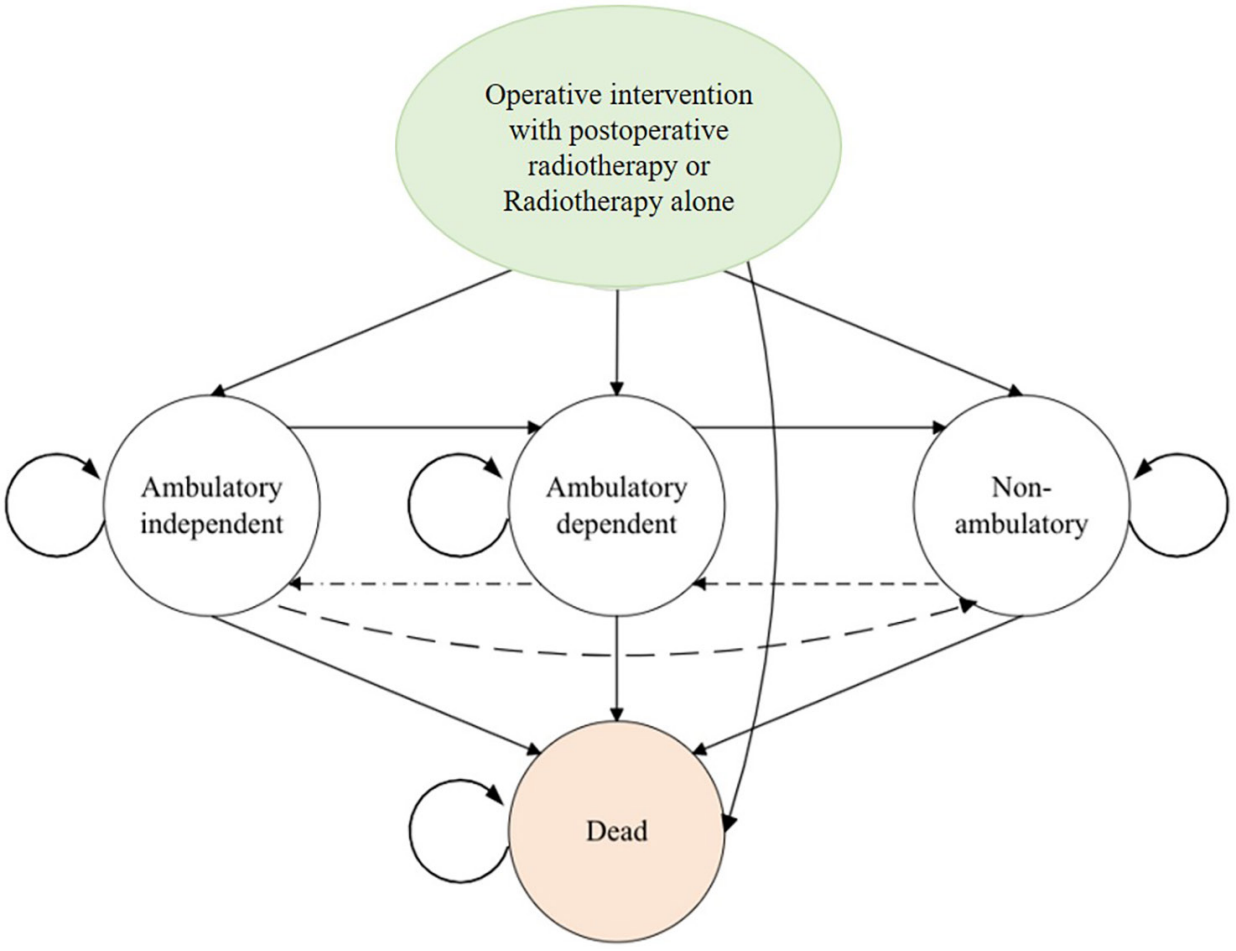
The structure of the Markov model compares operative intervention with postoperative radiotherapy and radiotherapy alone for patients with spinal metastases. The circles represent the health states. A simulated patient starts in the model at the time of initial presentation and undergoes an operative intervention with postoperative radiotherapy or radiotherapy alone (green circle). Following treatment, the patient may proceed to an ambulatory independent, ambulatory dependent, or nonambulatory state (white circles) or die (orange circle). At 30‐day intervals, patients may proceed to a different health state (straight arrow) or remain in their current health state (curved arrow). Due to the nature of epidural compression in those treated nonoperatively, transition from independent ambulatory to nonambulatory health states was modeled (longer dashed curved arrow). Transition from dependent to independent ambulatory health states was allowed in the first 6 months (dash‐dot arrow). Transition from nonambulatory function to dependent ambulatory function was only modeled in the operative arm (dashed arrow).

We constructed Markov state‐transition models simulating the therapeutic effect of either operative and radiotherapy management, and the model runs a monthly cycle. The patients' post‐treatment course was illustrated using a series of health‐state transitions over their remaining life expectancy (Figure 1). The transition probabilities, costs, and utility values were derived from either our institutions' or secondary data. Expenditures associated with the resources for patients of each health state are considered costs. The four‐stated Markov model is characterized by ambulatory function. The four states are defined as independent, dependent (requiring use of an assistive device), nonambulatory (bed or wheelchair‐bound), or death.^13^, ^19^, ^22^ We considered the efficacy of treatments as the probability to maintain or return ambulatory. The models' time frames were prespecified at 5 years for population A and 6 months for population B, considering their different life expectancies, or until all simulated patients died, whichever occurred first. TreeAge was used for statistical analysis.

### 2.7 Sensitivity and subgroup analyses

A third population, namely population C, who can walk independently at presentation with 3‐month survival probability >50%, was also simulated as a subgroup analysis (Table 1). The cost‐effectiveness of operative treatment changes was evaluated among different clinical symptoms, surgical procedures, survival probability cut points (30% and 70%), complication rates, and operative efficacy. We considered two additional procedures: posterior decompression with and without fusion to consider variation in the surgical types performed for spinal metastases. Considering some estimations and probabilities were derived from relatively small samples, we performed a probabilistic sensitivity analysis to evaluate the robustness of the study.^33^ The analyses were conducted by simulating various estimates of the inputs in 100,000 simulations and were illustrated using cost‐effectiveness acceptability curves to display the percentage of simulations where an operative or radiotherapy strategy was considered cost‐effective.

**TABLE 1:** Input parameters for transition probabilities, complications, and mortality for patients undergoing operative and nonoperative treatment in this cost‐effective analysis.^14^, ^16^, ^17^, ^19^, ^20^, ^22^, ^23^, ^24^, ^25^, ^27^

	3‐month >50%^a^		3‐month <50%^a^		Independent ambulatory and 3‐month >50%^a^	
	Operative (%)	Nonoperative (%)	Operative (%)	Nonoperative (%)	Operative (%)	Nonoperative (%)
First month						
Ind	27.0	38.7	27.0	38.7	27.9	40.8
Dep	43.4	28.0	43.4	28.0	41.2	29.4
NA	26.6	30.8	26.6	30.8	27.9	27.3
Mort	3.0	2.5	3.0	2.5	3.0	2.5
Complication	51	6.9	51	6.9	51	6.9
Transition probabilities						
Ind–Dep	2.9	1.8	2.8	1.8	2.9	1.8
Ind–NA	–	0.6	–	0.6	–	0.6
Ind–Mort	1.3	0.9	1.8	1.3	1.1	1.2
Dep–Ind	4.6^b^	1.2^b^	4.6^b^	1.2^b^	4.7^b^	1.2^b^
Dep–NA	1.1	1.9	1.1	1.8	1.1	1.8
Dep–Mort	2.9	2.0	4.3	3.0	2.6	2.8
NA–Ind	–	–^c^	–	–^c^	–	–^c^
NA–Dep	7.8^b^	–^c^	7.3^b^	–^c^	7.8^b^	–^c^
NA–Mort	9.8	6.9	14.3	10.0	8.9	9.4

Abbreviations: Dep, dependent ambulatory status; Ind, independent ambulatory status; Mort, mortality; NA, nonambulatory status.

^a^ The values are given as the rate.

^b^ These values are only applied for the first 6 months.

^c^ An assumption has been made that radiation cannot cause someone to improve from nonambulatory status.

## 3 RESULTS

### 3.1 Population A: 3‐month survival probability >50%

The accumulative QALY lifetime effectiveness was 1.31 (95% confidence interval [CI], 0.80–1.81; Table 2) after operative intervention with postoperative radiotherapy and 1.04 (95% CI, 0.63–1.43) after radiotherapy alone. The lifetime medical costs were $128,670 (95% CI, 121,485–136,152) after operative intervention with postoperative radiotherapy and $89,890 (95% CI, 83,745–96,496) for those who received radiotherapy alone. The ICER for operative intervention over radiotherapy was $140,907 per QALY.

**TABLE 2:** Cost‐effectiveness of nonoperative treatment and operative treatment in patients with independent ambulatory and nonambulatory status at presentation.

	Lifetime cost	QALY	ICER
3‐month survival probability >50%			
Operative treatment	$128,670 (121,485–136,152)	1.31 (0.80–1.81)	–
Nonoperative treatment	$89,890 (83,745–96,496)	1.04 (0.63–1.43)	$140,907
3‐month survival probability <50%			
Operative treatment	$61,988 (61,363–62,540)	0.24 (0.15–0.32)	–
Nonoperative treatment	$32,199 (31,682–32,652)	0.23 (0.14–0.31)	$3,178,510
Independent ambulatory at presentation with 3‐month survival probability >50%			
Operative treatment	$125,345 (118,198–132,775)	1.27 (0.76–1.76)	–
Nonoperative treatment	$84,160 (77,882–91,079)	1.02 (0.62–1.43)	$168,385

Abbreviations: ICER, incremental cost‐effectiveness ratio; QALY, Quality‐adjusted life year.

### 3.2 Population B: 3‐month survival probability <50%

The accumulative QALY lifetime effectiveness was 0.24 (95% CI, 0.15–0.32) after operative intervention with postoperative radiotherapy and 0.23 (95% CI, 0.14–0.31) after radiotherapy alone. The lifetime medical costs were $61,988 (95% CI, 61,363–62,540) after operative intervention with postoperative radiotherapy and $32,199 (95% CI, 31,682–32,652) for those who received radiotherapy alone. The ICER for operative intervention over radiotherapy was $3,178,510 per QALY.

### 3.3 Population C: Independent ambulatory at presentation with 3‐month survival probability >50%

The accumulative QALY lifetime effectiveness was 1.27 (95% CI, 0.76–1.76) after operative intervention with postoperative radiotherapy and 1.02 (95% CI, 0.62–1.43) after radiotherapy alone. The lifetime medical costs were $125,345 (95% CI, 118,198–132,775) after operative intervention with postoperative radiotherapy and $84,160 (95% CI, 77,882–91,079) for those who received radiotherapy alone. The ICER for operative intervention over radiotherapy was $168,385 per QALY.

### 3.4 Sensitivity analyses

The ICERs of operative intervention with postoperative radiotherapy, compared with radiotherapy alone, were $12,372 for patients with 3‐month survival probability >70%, $160,438 for patients with 3‐month survival probability >30%, $181,723 for patients with 3‐month survival probability <70%, and $210,168 for patients with 3‐month survival probability <30%. Regardless of applied cut points, an operative intervention is more cost‐effective for patients with better prognoses, and radiotherapy treatment is a more economical choice for patients with worse prognoses. For patients with independent ambulatory status, the ICERs were $148,685 when using 70% as cutoff and $192,337 when using 30% as cutoff. We also considered two additional surgical procedures as sensitivity analyses, and the surgical costs ranged from $32,118 to $71,688. In population A, at the WTP threshold of $150,000 per QALY, surgical interventions with postoperative radiotherapy were only cost‐effective when the costs were less than $40,500 (3.1 times the cost of radiotherapy).

**FIGURE 2:**
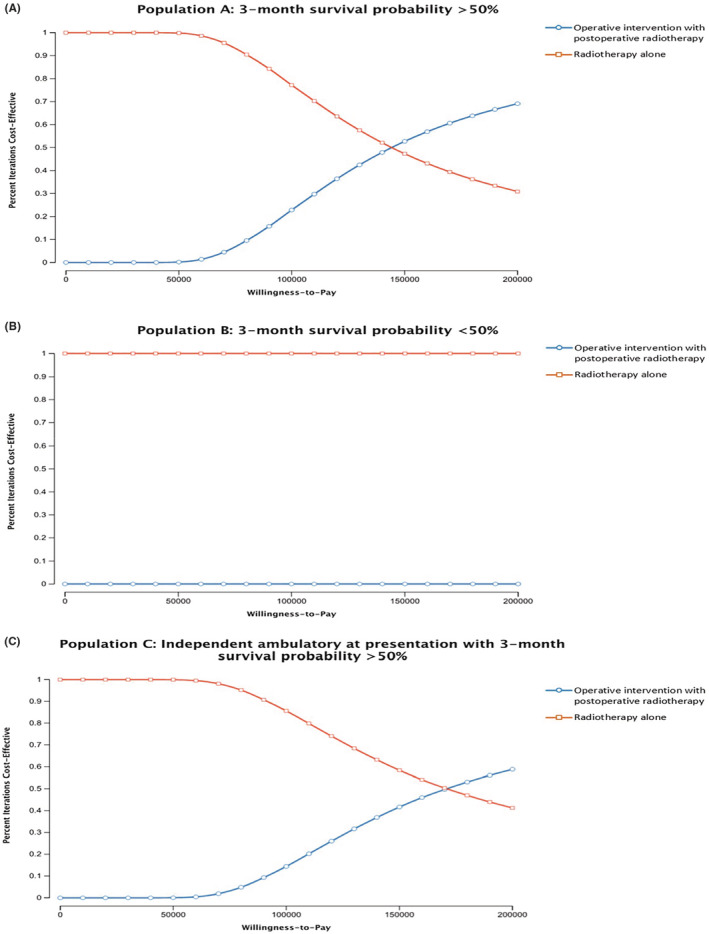
(A–C) Cost‐effectiveness acceptability curve for (A) patients with 3‐month survival probability >50%, (B) patients with 3‐month survival probability >50%, and (C) patients with independent ambulatory at presentation with 3‐month survival probability >50%, indicate the probability of operative intervention with postoperative radiotherapy or radiotherapy alone being the cost‐effective strategy at a given willingness to pay threshold.

In probabilistic sensitivity analysis (Figure 2), if the patients were willing to pay $100,000 for a QALY, the surgical procedure was cost‐effective in 23% of the simulations for population A, 0% for population B, and 14% for population C. At the WTP threshold of $150,000 per QALY, a surgical procedure was cost‐effective in 53% of the simulations for population A, 0% for population B, and 42% for population C.

## 4 DISCUSSION

In this study, we simulated the medical cost and gained utility of surgical intervention for spinal metastasis compared with radiotherapy. Following the survival predictions of SORG‐MLA, we found that surgical intervention is equivocally cost‐effective for patients with better prognoses but not cost‐effective for their counterparts. Although surgery was not an economical choice for population C, it became equivocally cost‐effective when setting the cutoff of 3‐month survival probability at 70%. We, therefore, recommend the SORG‐MLA for preoperative evaluation, as we demonstrated its clinical utility in regard of medical economics. With increasing health costs in our society, the importance of comparing operative treatment versus nonoperative treatment such as radiotherapy according to patient features is of value to our society.

Ideally, we should have tested the cost‐effectiveness of surgical interventions following all existing SPMs' prediction.^5^, ^6^, ^34^ However, we chose the SORG‐MLA, the most modern SPM, since previous studies reported that it outperformed the others in terms of discriminatory ability and overall performance.^1^, ^5^ The clinical cutoff of 50% might be arbitrary as modern orthopedic oncologists believed the clinical decision should be based on multidisciplinary decision.^11^, ^35^, ^36^ Also, the SORG‐MLA tended to underestimate the survival of Taiwanese patients.^1^, ^3^, ^11^ To avoid bias, we tested multiple cutoffs in sensitivity analyses and showed that SORG‐MLA was still clinically useful. When applying 50% and 70% as cut points, a surgical intervention was more cost‐effective for the patients with better predicted survival probabilities while not cost‐effective for the counterpart. These consistent results illustrated the importance of carefully selecting patients, example, patients with better prognosis, before they undergo surgery in regard to medical economics.

Schoenfeld et al.^17^ recently stratified patients based on clinical symptoms and reported that a surgical intervention is only cost‐effective for patients with non‐ambulation at presentation due to acute spinal cord compression. The rationale is that those confined to bed might regain their walking ability after decompression surgery (ICER, $48,600) while the incremental utility was relatively low for those with intact ambulation (ICER, $899,700). However, previous literature suggested that patients with better ambulatory ability usually had better prognoses.^1^, ^35^, ^36^ The aforementioned concepts might give contradictory clinical suggestions in the regard of medical economics. A clinician might have a hard time to decide a suitable treatment for a patient with intact walking ability (economically suitable for radiotherapy) and a long life expectancy (economically suitable for surgery). In order to resolve the discrepancy, we further analyzed a patient subgroup (population C). The ICER was much lower than the previously reported one^17^ without patient selection based on predicted survival (ICER, $899,700). The ratio could be further lower when selecting a higher survival probability as threshold. These results might highlight the importance of a pretreatment survival estimation as well as the importance of a precise and reliable SPM.

The neurologic, oncologic, mechanical, and systemic (NOMS) framework illustrates the modern concept that the clinical decision for patients with spinal metastases should be based on multidisciplinary decision.^37^ In this framework, patients with radioresistant tumors, mechanical instability, or acute neurological symptoms should be considered for a separation surgery and/or stabilization.^38^, ^39^ In contrast, patients without myelopathy, unstable structure, or previous radiation history are more suitable candidates for radiotherapy. In this study, we demonstrated the four mainstays could be evaluated together by stratifying patients into more subgroups (e.g., independent ambulatory at presentation [neurologic] with a 3‐month survival probability >50% [systemic]). Ideally, a future multicentered, prospective study with a large enough patient group could demonstrate the cost‐effectiveness of state‐of‐the‐art treatments in more detailed patient subgroup.

Cost‐effective analysis should be applied to each patient subgroup to guide clinical decision individually. Multiple factors, including clinical symptoms, disease course (e.g., whether metachronous or synchronous, whether previously using molecular treatment or not), survival probability, number of spinal metastatic lesions, and risk of vertebral fracture in the future, could be used to stratify the patient group, as they impact therapeutic effectiveness and costs.^39^, ^40^, ^41^, ^42^, ^43^ However, given the limited number of patients and complexity of metastatic disease, it would be impractical to consider all these factors in a single study. Therefore, only one^16^ of the four previous cost‐effective studies stratified patients into two subgroups, while the other studies did not perform subgroup analysis.^23^, ^44^, ^45^ In this current study, we considered patient's primary tumor type, clinical symptoms, number of spinal metastatic lesions, laboratory values, whether previously using molecular treatment or not, and combined these factors into a single index to get more personalized evaluation. This analysis should be more clinically appreciable, especially in such a complicated field, as it took more factors into account than prior researchers did.

This study had several limitations. First, many data were derived from retrospective cohorts, and selection or surveillance bias could hardly be avoided. We therefore used probabilistic sensitivity analysis to address this to the fullest extent possible. Moreover, the mortality rates of both operative and radiotherapy subgroups in this cohort were consistent with previous reports.^13^, ^46^, ^47^ We believed this might demonstrate the generalizability of our results and conclusions. Second, we did not consider the cost of postoperative chemotherapy, immunotherapy, or other molecular treatments. The medical treatments were effective but often expensive. These would certainly affect the health‐care expenditures and the therapeutic effectiveness. However, given the lack of detailed information, we were unable to conduct such an analysis. Third, while comparing the cost‐effectiveness of surgery versus EBRT, it is important to consider stereotactic body radiotherapy (SBRT) as an alternative for ambulatory patients with metastatic epidural canal compromise from radiosensitive tumors and stable spinal conditions. SBRT has shown superiority over EBRT in pain relief and local tumor control,^48^, ^49^, ^50^ potentially narrowing the cost‐effectiveness difference between surgery and radiotherapy. However, only 7.2% (200/2768) of patients received SBRT, presenting difficulties in executing a stratified analysis within this study. As the utilization of SBRT increases, upcoming studies could delve into the cost‐effectiveness aspects of surgery, EBRT, and SBRT to offer insights for clinical decision‐making. Fourth, due to ethical issues, it is impossible to conduct a randomized trial to evaluate the quality of life, pain, and ambulatory function after operative and nonoperative treatments. We have to admit that patients who underwent surgical procedure might have favorable oncological and clinical characteristics. Therefore, these findings were not applicable to clinical situations when considering a palliative treatment. Fifth, to conduct this cost‐effective analysis, we had to dichotomize the survival probability and set default therapeutic strategy based on the prediction. This method might be against the modern concept that treatment plans should be based on multidisciplinary decision. The dilemma could only be solved by stratifying patients into more detailed subgroups using a large enough cohort. Lastly, it would be of clinical interest to perform cost‐effective analysis to patients with non‐ambulation and 3‐month survival probability <50%, as the two characteristics might also give contradictory therapeutic suggestions. However, we failed to estimate the therapeutic efficacy of operation treatment since comprehensive data were lacking. Despite these limitations, we demonstrated that neurological symptoms and systematic conditions could be evaluated together. We also reported that decompression surgery could be cost‐effective for patients with intact ambulatory function, especially for those with great prognosis (3‐month survival probability >70%).

With increasing health costs in our society, both clinical gain as well as value to society are important when elucidating the optimal candidate for surgical intervention. Patient selection based on survival estimation was highlighted in this study. A surgical procedure might be cost‐effective in subgroups such as patients with better survival and intact ambulatory ability. Future research using larger databases should provide more stratified results by indication and clinical characteristics.

## AUTHOR CONTRIBUTIONS

**Hsiang‐Chieh Hsieh:** Conceptualization (equal); writing – original draft (equal). **Hung‐Kuan Yen:** Data curation (equal); writing – original draft (equal). **Ting‐En Tseng:** Conceptualization (equal); writing – original draft (equal). **Yu‐Ting Pan:** Data curation (equal); writing – original draft (equal). **Min‐Tsun Liao:** Methodology (equal); supervision (equal). **Shau‐Huai Fu:** Conceptualization (equal); writing – review and editing (equal). **Mao‐Hsu Yen:** Formal analysis (equal); supervision (equal). **Fu‐Shan Jaw:** Methodology (equal); supervision (equal). **Wei‐Hsin Lin:** Methodology (equal); supervision (equal). **Ming‐Hsiao Hu:** Conceptualization (equal); supervision (equal). **Shu‐Hua Yang:** Supervision (equal). **Olivier Q. Groot:** Conceptualization (equal); writing – review and editing (equal). **Andrew J. Schoenfeld:** Conceptualization (equal); supervision (equal).

## FUNDING INFORMATION

National Taiwan University Hospital, Taipei, Taiwan.

## CONFLICT OF INTEREST STATEMENT

Each author certifies that there are no conflicts of interest.

## Supporting information


Table S1

## Data Availability

Data sharing is not applicable to this article as no new data were created or analyzed in this study.
