# Supplementary material for: Determining patients with spinal metastases suitable for surgical intervention: A cost‐effective analysis
Source: Cancer Med. 2023 Sep 25;12(19):20059–69. doi: 10.1002/cam4.6576 (PMC10587930; doi:10.1002/cam4.6576)
Supplement: Supplementary file 1 — Table S1 [file CAM4-12-20059-s001.docx]

**Supplementary Table 1**. Cost, utility, and the Markov state-transition model

| **Population A Operative Treatment** | | **Population A Nonoperative Treatment** | |
| --- | --- | --- | --- |
| Complication: 51%^22^  SSI: 5.1%^14^  Other: 94.9%  Mort: 3%^22^  Revision Surgery: 15%^17^  No complication: 8%^22^  SSI: 11%^20^  Other: 11%^20^ | | Complication: 6.9%^23^  VF: 3.6%^27^  Other: 96.4%  Mort: 2.5%**  Primary Surgery: 30%^17^  No complication: 0.8%^24^  VF: 4.8%^25^  Other: 11%^20^ | |
| Transition Probabilities | | Transition Probabilities | |
| First month^16, 19^  $\begin{matrix} Ind & 27.8\% \\ Dep & 44.7\% \\ NA & 27.5\% \end{matrix}$ | Each Addition Month^19^  $\begin{matrix} Ind & Dep & NA & Mort \\ 97.1\% & 2.9\% & -- & 2.8\%*** \\ 4.8\%* & 94.1\% & 1.1\% & 6.5\%*** \\ -- & 8.6\%* & 91.4\% & 21.9\%*** \end{matrix}$ | First month^16, 19^  $\begin{matrix} Ind & 39.7\% \\ Dep & 28.7\% \\ NA & 31.6\% \end{matrix}$ | Each Addition Month^19^  $\begin{matrix} Ind & Dep & NA & Mort \\ 97.6\% & 1.8\% & 0.6\% & 2.8\%*** \\ 1.2\%* & 96.9\% & 1.9\% & 6.5\%*** \\ -- & -- & 100\% & 21.9\%*** \end{matrix}$ |
| **Population B Operative Treatment** | | **Population B Nonoperative Treatment** | |
| Complication: 51%^22^  SSI: 5.1%^14^  Other: 94.9%  Mort: 3%^22^  Revision Surgery: 15%  No complication: 8%^22^  SSI: 11%^20^  Other: 11%^20^ | | Complication: 6.9%^23^  VF: 3.6%^27^  Other: 96.4%  Mort: 2.5%**  Primary Surgery: 30%  No complication: 0.8%^24^  VF: 4.8%^25^  Other: 11%^20^ | |
| Transition Probabilities | | Transition Probabilities | |
| First month^16, 19^  $\begin{matrix} Ind & 27.8\% \\ Dep & 44.7\% \\ NA & 27.5\% \end{matrix}$ | Each Addition Month^19^  $\begin{matrix} Ind & Dep & NA & Mort \\ 97.1\% & 2.9\% & -- & 2.8\%*** \\ 4.8\%* & 94.1\% & 1.1\% & 6.5\%*** \\ -- & 8.6\%* & 91.4\% & 21.9*** \end{matrix}$ | First month^16, 19^  $\begin{matrix} Ind & 39.7\% \\ Dep & 28.7\% \\ NA & 31.6\% \end{matrix}$ | Each Addition Month^19^  $\begin{matrix} Ind & Dep & NA & Mort \\ 97.6\% & 1.8\% & 0.6\% & 2.8\%*** \\ 1.2\%* & 96.9\% & 1.9\% & 6.5\%*** \\ -- & -- & 100\% & 21.9*** \end{matrix}$ |
| **Population C Operative Treatment** | | **Population C Nonoperative Treatment** | |
| Complication: 51%^22^  SSI: 5.1%^14^  Other: 94.9%  Mort: 3%^22^  Revision Surgery: 15%^17^  No complication: 8%^22^  SSI: 11%^20^  Other: 11%^20^ | | Complication: 6.9%^23^  VF: 3.6%^27^  Other: 96.4%  Mort: 2.5%**  Primary Surgery: 30%^17^  No complication: 0.8%^24^  VF: 4.8%^25^  Other: 11%^20^ | |
| Transition Probabilities | | Transition Probabilities | |
| First month^19^  $\begin{matrix} Ind & 28.7\% \\ Dep & 42.5\% \\ NA & 28.7\% \end{matrix}$ | Each Addition Month^19^  $\begin{matrix} Ind & Dep & NA & Mort \\ 97.1\% & 2.9\% & -- & 2.8\%*** \\ 4.8\%* & 94.1\% & 1.1\% & 6.5\%*** \\ -- & 8.6\%* & 91.4\% & 21.9*** \end{matrix}$ | First month^19^  $\begin{matrix} Ind & 41.8\% \\ Dep & 30.2\% \\ NA & 28.0\% \end{matrix}$ | Each Addition Month^19^  $\begin{matrix} Ind & Dep & NA & Mort \\ 97.6\% & 1.8\% & 0.6\% & 2.8\%*** \\ 1.2\%* & 96.9\% & 1.9\% & 6.5\%*** \\ -- & -- & 100\% & 21.9*** \end{matrix}$ |
| **Cost^1, 2, 15, 18, 26, 28, 29, 30^** | | | **Utility^29^** |
| Health State  $\begin{matrix} Ind & \$2,407 \\ Dep & \$2,542 \\ NA & \$5,582 \end{matrix}$ | Event  $\begin{matrix} SSI & VF & Become Dep & Become NA \\ \$7,780 & \$2,040 & \$53 & \$94 \end{matrix}$ | Treatment  $\begin{matrix} Surgery & \$33,529 \\ Rev. Surg & \$7,405 \\ Radiation & \$12,932 \end{matrix}$ | Health State  $\begin{matrix} Ind & 0.756 \\ Dep & 0.599 \\ NA & 0.175 \end{matrix}$ |

*Designates a value that is only applied for the first 6 months; it then converts to 0. The complement probability to stay in the respective health state is thus increased to ensure the sum remains at 1.

** Designates an assumption made based on operative overall mortality

*** These probabilities were adjusted for cohort-specific mortality while the ratio remained the same.

+ Designates an assumption made that radiation cannot cause someone to improve from non-ambulatory status.

^ If non-ambulatory and the number of months is <8, this revision surgery/primary surgery probability is used.

Mort = Mortality

Ind = Independent
